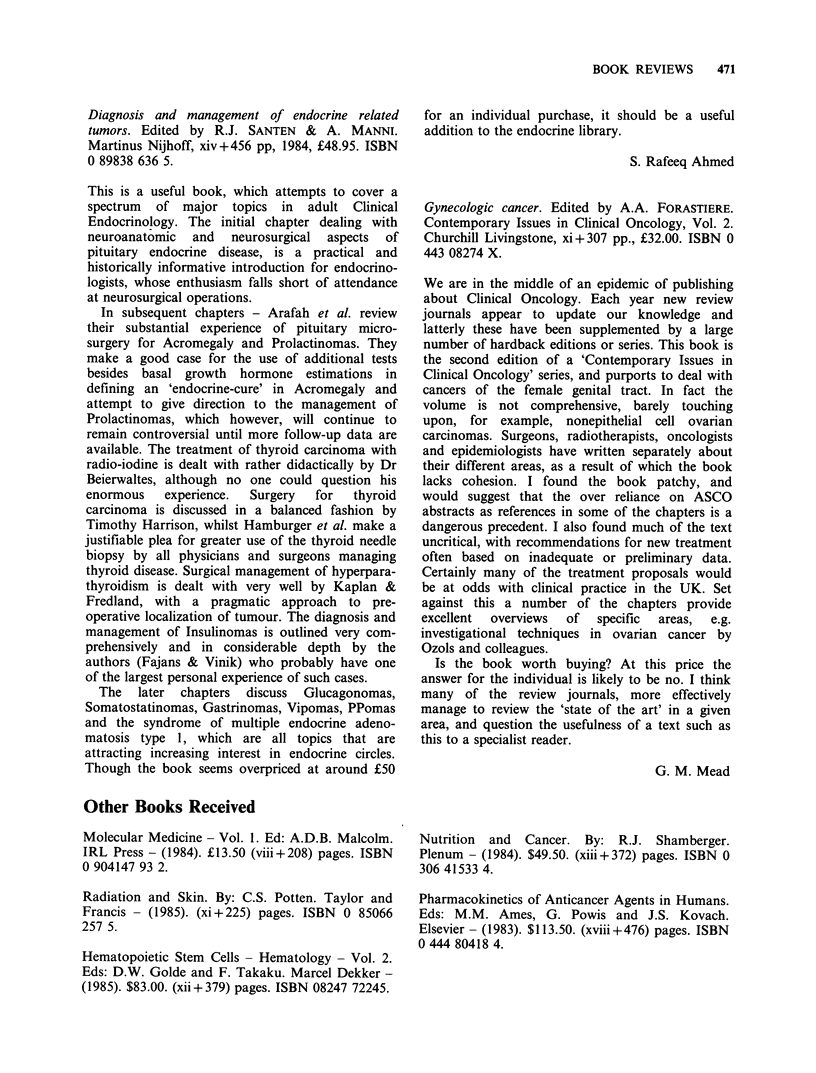# Diagnosis and management of endocrine related tumors

**Published:** 1985-09

**Authors:** S. Rafeeq Ahmed


*Diagnosis and management of endocrine related tumors*. Edited by R.J. SANTEN & A. MANNI. Martinus Nijhoff, xiv+456 pp, 1984, £48.95. ISBN 0 89838 636 5.

This is a useful book, which attempts to cover a spectrum of major topics in adult Clinical Endocrinology. The initial chapter dealing with neuroanatomic and neurosurgical aspects of pituitary endocrine disease, is a practical and historically informative introduction for endocrinologists, whose enthusiasm falls short of attendance at neurosurgical operations.

In subsequent chapters – Arafah *et al.* review their substantial experience of pituitary microsurgery for Acromegaly and Prolactinomas. They make a good case for the use of additional tests besides basal growth hormone estimations in defining an 'endocrine-cure' in Acromegaly and attempt to give direction to the management of Prolactinomas, which however, will continue to remain controversial until more follow-up data are available. The treatment of thyroid carcinoma with radio-iodine is dealt with rather didactically by Dr Beierwaltes, although no one could question his enormous experience. Surgery for thyroid carcinoma is discussed in a balanced fashion by Timothy Harrison, whilst Hamburger *et al.* make a justifiable plea for greater use of the thyroid needle biopsy by all physicians and surgeons managing thyroid disease. Surgical management of hyperparathyroidism is dealt with very well by Kaplan & Fredland, with a pragmatic approach to preoperative localization of tumour. The diagnosis and management of Insulinomas is outlined very comprehensively and in considerable depth by the authors (Fajans & Vinik) who probably have one of the largest personal experience of such cases.

The later chapters discuss Glucagonomas, Somatostatinomas, Gastrinomas, Vipomas, PPomas and the syndrome of multiple endocrine adenomatosis type 1, which are all topics that are attracting increasing interest in endocrine circles. Though the book seems overpriced at around £50 for an individual purchase, it should be a useful addition to the endocrine library.

S. Rafeeq Ahmed